# Research on the construction of prediction model for depressive symptom in the second and third trimester of pregnancy based on artificial neural network

**DOI:** 10.3389/fpsyt.2026.1792277

**Published:** 2026-05-22

**Authors:** Liuyue Wang, Dandan Zhou, Yanhui Liu, Zhiqun Liu, Huan Wan

**Affiliations:** 1 Nursing Department, The First Affiliated Hospital of Hunan Normal University (Hunan Provincial People’s Hospital), Changsha, Hunan, China; 2 Nursing College, School of Medicine, Hunan Normal University, Changsha, Hunan, China; 3 Kiang Wu Nursing College of Macau, Macao, SAR, China; 4 School of Nursing, Changsha Medical College, Changsha, Hunan, China; 5 Nursing Department, Hunan Provincial People’s Hospital (The First Affiliated Hospital of Hunan Normal University), Changsha, Hunan, China; 6 Department of Emergency Medicine, Clinical Research Center For Emergency and Critical Care In Hunan Province, Hunan Provincial Institute of Emergency Medicine, Hunan Provincial Key Laboratory of Emergency and Critical Care Metabonomics, Hunan Provincial People's Hospital, The First Affiliated Hospital of Hunan Normal University, Changsha, Hunan, China; 7 Department of Psychology, College of Education Science, Hunan Normal University, Changsha, Hunan, China

**Keywords:** artificial neural network, depression, pregnancy, psychological nursing, risk prediction

## Abstract

**Objective:**

To investigate the current status of depression in women in the second and third trimesters of pregnancy, analyze the factors associated with depressive symptoms during this period,and construct an artificial neural network-based risk prediction model for depressive symptoms, with the aim of providing evidence for early screening, risk identification, and targeted intervention.

**Methods:**

A convenience sample of 588 women in the second and third trimesters of pregnancy (28 -36 weeks of pregnancy) who had regular prenatal check-ups in the obstetric outpatient clinics of two tertiary hospitals in Changsha and Hengyang from April 2023 to December 2023 was investigated using the questionnaire for depressive symptoms in the second and third trimesters of pregnancy.Spearman correlation analysis was used to explore the correlation between social support, coping style, vulnerable personality, parenting competence and the Edinburgh postpartum depression score. Binary logistic regression and a multi-layer perceptron artificial neural network were used to construct prediction models for depressive symptoms in the second and third trimesters, respectively.

**Results:**

The positive rate of depressive symptoms screening in the second and third trimesters was 42.7%.The level of social support, active coping and reactive personality were negatively correlated with depressive symptoms in the second and third trimesters of pregnancy (*P*< 0.05), while negative coping and vulnerable personality were positively correlated with depressive symptoms in the second and third trimesters of pregnancy (*P*< 0.05). The prediction accuracy of the logistic regression model for predicting depressive symptoms in the second and third trimesters of pregnancy was 79.6%, and the prediction accuracy of the risk prediction model for depressive symptoms in the second and third trimesters of pregnancy based on artificial neural networks was 86.9%.

**Conclusion:**

The positive rate of depression symptom screening is higher in women during the second and third trimesters. Nursing measures can be formulated based on the associated factors in this model to support early identification and targeted intervention for depressive symptoms in the second and third trimesters of pregnancy. The prediction model for depression symptoms in the second and third trimester constructed in this study has good predictive performance and can provide a basis for clinical nurses to identify women at high risk of depressive symptoms.

## 1 Introduction

Perinatal depression (PND) refers to a mood disorder that occurs in women from the 28th week of pregnancy to one week after delivery, accompanied by symptoms such as low mood, loss of interest, decreased attention, depressed mood, and low self-esteem. It is the most common mood disorder in women during pregnancy (1). Peripartum women are at higher risk of depression than the general population due to factors such as large fluctuations in estrogen and progesterone levels, body changes, and role transitions. The incidence of depression is highest in the second and third trimesters and one week after delivery (2). However, the mental health problems of pregnant womenare often neglected by pregnant women and their families. Few women actively seek help when they experience depressive symptoms (3). This may contribute to delayed identification and intervention for PND, which is associated with adverse maternal and infant outcomes, and has a long-term negative impact on the health of pregnant women themselves and their offspring, and may even be associated with severe consequences such as suicide or infanticide. Therefore, the mental health of perinatal women needs to be taken seriously.

However, perinatal depression is not solely related to physiological and social factors such as hormonal fluctuations, physical changes, and role transitions. It also involves individual psychological vulnerability, emotional regulation abilities, and coping mechanisms during the perinatal period (4). Existing research suggests that prenatal depression is associated with emotional vulnerability factors such as low self-esteem, lower maternal responsiveness, high perfectionism, and higher levels of anger. It may also be associated with adverse fetal and maternal outcomes through elevated maternal cortisol levels and alterations in the hypothalamic-pituitary-adrenal axis. Recent research based on a sample of Italian pregnant women further indicates that psychological factors may have more significant predictive value than sociodemographic factors (5). Simultaneously, low social support has been shown to be significantly associated with prenatal depression (6). Therefore, exploring the related factors of perinatal depression from psychosocial dimensions such as social support, coping styles, and vulnerable personality traits has important theoretical and practical significance.

Currently, there are relatively few studies on the prediction of depressive symptoms in the second and third trimesters, and most of them use traditional logistic regression models for prediction. Hu et al. (7) constructed a multifactorial stepwise logistic regression model for perinatal depression in late pregnancy based on prenatal social and psychological factors. The model showed high prediction accuracy and has certain guiding significance for perinatal depression screening in primary hospitals. However, the model has a low specificity for depression screening in women in late pregnancy.

International studies on the prediction of depressive symptoms in the second and third trimesters of pregnancy are relatively innovative in their methods. Most of them select the optimal prediction model by comparing multiple models. Dayeon (8) tried to use machine learning methods such as Naive Bayes and Support Vector Machine to build a postpartum depression prediction model, and compared the prediction effects of various machine learning methods. Although the above studies can select the model with the highest prediction accuracy, they have not focused on the prediction of depressive symptoms in the second and third trimesters of pregnancy and may have limited value for early identification and intervention during pregnancy. Therefore, it is still necessary to explore prediction models with high prediction accuracy, which can identify the risk of depressive symptoms in the second and third trimesters of pregnancy and are suitable for rapid screening of large samples.

Artificial Neural Network (ANN) refers to a mathematical model that simulates the processing of information by human brain neurons by artificially constructing a network to imitate the brain’s processing of external information. Through deep learning, computers can recognize data, images, audio and other information to support prediction (9, 10). ANN can build medical or nursing models through a variety of algorithms to predict the interaction between dependent variables and independent variables. The prediction model constructed may have high accuracy and sensitivity (11). ANN can effectively and scientifically process and analyze complex nonlinear relationships in the model, and is especially suitable for analyzing nonlinear relationships related to changes in patients’ mental states (12).

This study intends to combine artificial neural networks and logistic regression to construct a prediction model for depressive symptoms in mid- and late pregnancy, screen people at high risk of depressive symptoms in mid- and late pregnancy, and provide a basis for clinical implementation of targeted and efficient psychological care for pregnant women.

## 2 Objects and methods

### 2.1 Research subjects

Pregnant women in the second and third trimesters of pregnancy(28-36 weeks of gestation) who received regular prenatal checkups in the obstetrics clinics of a tertiary hospital in Changsha and a tertiary hospital in Hengyang from April 2023 to December 2023 were selected as research subjects.Inclusion criteria: Pregnant women in the second and third trimesters of pregnancy(28-36 weeks of gestation), conscious and able to communicate without difficulty, and willing to participate in this study after providing informed consent. Exclusion criteria: Pregnant women with insufficient reading comprehension skills who were unable to complete the questionnaire.

This study used PASS15.0 software to calculate the sample size, setting the type I error α of the hypothesis test to 0.05 and the allowable error (δ) to 0.03. A literature review indicates that the positive rate for mid-to-late pregnancy depressive symptom screening is approximately 17%, calculated based on the sample size formula for cross-sectional studies:


$$\text{n}={\text{Z}}^{2\text{α}}\text{p}(1-\text{p})/{\text{δ}}^{2}$$

Calculations show that 459 cases are needed for the second and third trimesters survey. The data missing rate was set at 10%, so the final sample size required for the second and third trimesters was 575 cases. A total of 588 pregnant women completed the survey, meeting the minimum sample size requirement. This study was approved by the hospital ethics committee (2021-149).

### 2.2 Methods

#### 2.2.1 Survey tools

##### 2.2.1.1 General information questionnaire

It is self-designed and includes 27 items, including the demographic characteristics of pregnant women (age, occupation, education level, medical insurance type, etc.), pregnancy status (pregnancy plan, pregnancy complications, etc.), and family support (husband-wife relationship, mother-in-law and daughter-in-law relationship, etc.).

##### 2.2.1.2 Edinburgh postnatal depression scale

The scale has 10 items, scored on a scale of 0 (never), 1 (occasionally), 2 (frequently), and 3 (always). Items 1 and 2 are scored in reverse order, and items 3-10 are scored in forward order. The score range is 0-30 points, with higher scores indicating more severe depression (13). The EPDS is simple, easy to use, and quickly and effectively identifies patients with depression, and is the preferred scale for PND assessment recommended by the guidelines (14). Considering that the optimal cutoff value of EPDS is affected by language version, cultural background and perinatal stage, this study refers to the validation study of pregnant samples in mainland China and uses EPDS≥9 as the positive cutoff value for screening depressive symptoms (15). Previous studies have shown that the optimal cutoff value of EPDS in this population is about 9.5 points, with a sensitivity of 80.0% and a specificity of 83.03%, suggesting that this cutoff value is more suitable for improving screening sensitivity and reducing missed detection of high-risk individuals (16). At the same time, a large-scale meta-analysis at the individual level has shown that EPDS≥11 can achieve a good balance between sensitivity and specificity, while EPDS≥13 has higher specificity but relatively lower sensitivity (17). Therefore, the purpose of using a lower cutoff value in this study is mainly for early risk identification in the perinatal population, rather than clinical diagnosis. At present, this scale has been demonstrated to have high reliability and validity in both prenatal and postnatal use (18). In this study, the prenatal Cronbanch’α coefficient was 0.82.

##### 2.2.1.3 Social support rate scale

This scale, revised by Professor Xiao Shuiyuan, consists of 10 items, encompassing three dimensions: support utilization (questions 8, 9, and 10, 12 points), subjective support (questions 1, 3, 4, and 5, 20 points), and objective support (questions 2, 6, and 7, 32 points). The total score is the sum of the scores for all 10 items. A higher total score indicates a higher level of social support, with scores of 45 to 64 indicating high social support, 23 to 44 indicating moderate social support, and ≤22 indicating low social support. The Cronbanch’s ɑ coefficients for the three dimensions ranged from 0.78 to 0.80 (19).

##### 2.2.1.4 Simplified coping style questionnaire

The 20-item scale encompasses two dimensions: positive and negative coping styles. Scoring is based on a 1-4 Likert scale, with scores ranging from 0 (never adopting coping styles), 1 (occasionally adopting coping styles), 2 (sometimes adopting coping styles), and 3 (often adopting coping styles). The sum of scores for items 1-12 represents the positive dimension, while the sum of scores for items 13-20 represents the negative dimension. The Cronbanch’s ɑ coefficient for the total scale in this study was 0.84, with Cronbanch’s ɑ coefficients for the positive and negative dimensions being 0.77 and 0.87, respectively (20).

##### 2.2.1.5 Vulnerable personality style questionnaire

This study used a revised version of the questionnaire developed by Jin et al (21). Which consists of 9 items divided into two dimensions: susceptibility and reactivity. It was used to measure the personality traits of perinatal women. Each item was scored as 1 (not at all), 2 (not), 3 (so-so), 4 (so), and 5 (exactly so). In previous studies, the Cronbanch’ɑ coefficient of this scale was 0.65.

#### 2.2.2 Data collection methods

This study distributed questionnaires online through WJX. Before distributing the questionnaires, researchers from the multi-center group leader units provided training on questionnaire distribution to researchers from participating units and standardized the distribution procedures and questionnaire interpretation standards.

Before distributing the questionnaire, the researcher briefly explained the purpose and significance of the study to the participants and provided them with instructions for completing the questionnaire. After obtaining informed consent, the participants completed the questionnaire online by scanning a QR code on WeChat. The researcher answered any questions the participants had during the questionnaire-filling process. After the questionnaire was completed, the researcher collected it immediately. If the researcher discovered any missing items or irregularities in the completed questionnaire, the researcher promptly informed the participants to complete the questionnaire to ensure the validity and accuracy of the data collection.

To ensure the questionnaire response rate, the questionnaire survey was conducted when pregnant women were undergoing fetal heart monitoring examinations, which provided them with ample time and made it easier for them to cooperate.

#### 2.2.3 Statistical analysis

This study used SPSS 23.0 statistical software for analysis. Basic data of perinatal women were described using frequencies and percentages. The chi-square test and Fisher’s exact test were used to compare differences in the positive rate of depressive symptom screening among different basic characteristics. For correlation analysis, Pearson correlation analysis was used for normally distributed data, and Spearman correlation analysis was used for non-normally distributed data. A binary logistic regression analysis was used to construct a predictive model for mid-to-late pregnancy depressive symptoms, and the Hosmer–Lemeshow test was used to determine the goodness of fit of the predictive model. The significance level was set at α = 0.05, and *P<* 0.05 was considered statistically significant. A multilayer perceptron was used to construct a predictive model for depressive symptoms in mid-to-late pregnancy, and the importance of independent variables was determined. Receiver operating characteristic (ROC) curves were used to test the sensitivity and specificity of the model, and the area under the ROC curve (AUC) was used to evaluate discrimination.

#### 2.2.4 Methods for establishing a prediction model for mid-to-late pregnancy depression based on artificial neural networks

##### 2.2.4.1 Selection of modeling variables

Using the independent variables with significant differences identified by univariate tests as the input layer and the low, medium, and high risk levels of PND as the output layer, a predictive model for mid-to-late pregnancy depression was established.

##### 2.2.4.2 Modeling parameter settings

The ANN model parameters are set as follows: 80% of the samples are randomly selected from the training set, and 20% are randomly selected from the validation set. Five-fold cross-validation is used for model training and internal validation on the training set to optimize model parameters and reduce the risk of overfitting. Finally, the model’s predictive performance and generalization ability are evaluated on the validation set. There are two hidden layers with the hyperbolic tangent activation function, and the output layer activation function is Softmax. The learning rate is set to 0.01, the maximum number of iterations is set to 1000, the training type is batch processing, and the training termination rule is that training terminates when the maximum number of steps reaches 1 without reducing the prediction error. The maximum training time is 15 minutes.

#### 2.2.5 Quality control

Data collection for this study was conducted by a designated researcher, and subjects were screened strictly according to the inclusion and exclusion criteria. Returned questionnaires were checked for completeness. If any items were missing, incorrectly filled, or filled out inappropriately, the subjects were promptly contacted and the missing items were completed to avoid invalid questionnaires.

The data were entered and verified by two people. Parameters were set and debugged reasonably during the research process. The training termination rule was set as follows: when the error did not decrease in one consecutive step, training was stopped to reduce the risk of overfitting.

## 3 Results

### 3.1 Depression status in the second and third trimesters and univariate analysis results

The total score for women in the second and third trimesters was 7.97 ± 3.60, and the positive rate of depressive symptom screening in the second and third trimesters was 42.7%. Univariate analysis showed that a total of 13 variables were statistically significant (all *P*< 0.05), as shown in **Table 1** (only statistically significant parameters are listed).

**Table 1:** Univariate analysis of depression in the second and third trimesters.

Variable	Category	N		Positive rate (%)	χ²	*p*-value
		EPDS<9	EPDS≥9			
Place of Residence	Rural	21	35	62.5%	10.66^a^	0.005
	Town	28	24	46.1%		
	City	288	192	40.0%		
Education Level	Junior high or below	14	25	64.1%	9.09^a^	0.028
	High school/Technical	61	50	45.0%		
	Bachelor/Associate	222	153	40.8%		
	Graduate or above	40	23	36.5%		
Stable Job	No	95	102	51.8%	10.01^a^	0.002
	Yes	242	149	38.1%		
Medical Insurance	Rural Cooperative	71	213	75.0%	10.66^a^	0.014
	Urban Employee Basic	72	125	63.5%		
	Urban Resident Basic	25	27	51.9%		
	Self-pay	28	27	49.1%		
Monthly Family Income (RMB)	<3000	9	8	47.1%	25.73^a^	<0.001
	3000	38	67	63.8%		
	5000	139	96	40.9%		
	>10000	151	80	34.6%		
Recent Appetite	Average	69	110	61.5%	37.04^a^	<0.001
	Good	268	141	34.5%		
Marital Relationship	Average	10	37	78.72%	55.383^a^	<0.001
	Satisfied	123	131	51.6%		
	Very satisfied	204	83	28.9%		
Relationship with Mother-in-law	No need to handle	17	7	29.2%	64.63^a^	<0.001
	Average	35	86	71.1%		
	Satisfied	154	115	42.8%		
	Very satisfied	131	43	24.7%		
Personal Anxiety/ Depression History	No	327	215	39.7%	25.81^a^	<0.001
	Yes	10	36	78.3%		
Family Depression History	No	330	235	35.8%	7.07^a^	0.008
	Yes	7	16	69.6%		
Concern About Fetal Health	No	61	32	34.4%	3.10^a^	0.049
	Yes	276	219	44.2%		
Pregnancy Complications	No	279	190	40.5%	4.48^a^	0.034
	Yes	58	61	51.3%		
Fear of Childbirth	No	57	26	31.3%	5.10^a^	0.024
	Yes	280	225	44.6%		

### 3.2 Correlation between social support, coping style, and susceptible personality and depression in the second and third trimesters

Social support, coping style, vulnerable personality, and Edinburgh Postnatal Depression Scale (EPDS) scores were not normally distributed. Spearman correlation analysis showed that subjective support, objective support, support utilization, positive coping, and reactive personality were negatively correlated with EPDS scores(*P*< 0.01); negative coping and vulnerable personality were positively correlated with EPDS scores (*P*< 0.01). See **Table 2** for details.

**Table 2:** Correlation analysis between depression in the second and third trimesters and social support, coping style, and susceptible personality.

Variable	EPDS	Subjective support	Objective support	Support utilization	Positive coping	Negative coping	Vulnerable personality	Reactive personality
EPDS	1							
Subjective Support	-0.34**	1						
Objective Support	-0.29**	0.28**	1					
Support Utilization	-0.33**	0.36**	0.40**	1				
Positive Coping	-0.39**	0.39**	0.32**	0.49**	1			
Negative Coping	0.12**	0.05	-0.01	0.07	0.31**	1		
Vulnerable Personality	0.52**	-0.26**	-0.16**	-0.12**	-0.22**	0.28**	1	
Reactive Personality	-0.30**	0.30**	0.30**	0.43**	0.88**	0.23**	-0.19**	1

### 3.3 Multifactorial analysis of depression in the second and third trimesters of pregnancy

Logistic regression analysis was performed using the variables with statistical differences in the univariate analysis as independent variables and the presence or absence of depressive symptom as the dependent variable to establish a regression model for predicting depressive symptoms in the second and third trimesters.

Dummy variables were set for place of residence, education level, medical insurance type, marital relationship, and mother-in-law and daughter-in-law relationship during the second and third trimesters. The values of each variable are shown in **Table 3**.

**Table 3:** Variable assignments in the logistic regression analysis of depression in the second and third trimesters.

Variable	Coding
*Y:*Depression Status	No depression=0, Depression=1
*X_1_:*Place of Residence	City (Reference) *X_11_* = 0, *X_12_* = 0
	Rural(*X_11_*)*X_11_* = 1, *X_12_* = 0
	Town(*X_12_*)*X_11_* = 0, *X_12_* = 1
*X_2_:*Education Level	Graduate or above (Reference) *X_21_* = 0, *X_22_* = 0, *X_23_* = 0
	Junior high or below(*X_21_*)*X_21_* = 1, *X_22_* = 0, *X_23_* = 0
	High school/Technical secondary(*X_22_*)*X_21_* = 0, *X_22_* = 1, *X_23_* = 0
	Bachelor/Associate degree(*X_23_*)*X_21_* = 0, *X_22_* = 0, *X_23_* = 1
*X_3_:*Stable Job	No=0, Yes=1
*X_5_:*Medical Insurance Type	Self-pay (Reference) *X_51_* = 0, *X_52_* = 0, *X_53_* = 0
	Rural Cooperative Medical Scheme *X_51_* = 1, *X_52_* = 0, *X_53_* = 0
	Urban Employee Basic Medical Insurance *X_51_* = 0, *X_52_* = 1, *X_53_* = 0
	Urban Resident Basic Medical Insurance *X_51_* = 0, *X_52_* = 0, *X_53_* = 1
*X_6_:*Monthly Family Income (RMB)	<3000 = 1, 3000-5000 = 2, 5000-10000 = 3, >10000 = 4
*X_7_:*Recent Appetite	Average=1, Average=2
*X_8_:*Marital Relationship	Very satisfied (Reference) *X_81_* = 0, *X_82_* = 0, *X_83_* = 0
	Average *X_81_* = 0, *X_82_* = 1, *X_83_* = 0
	Satisfied *X_81_* = 0, *X_82_* = 0, *X_83_* = 1
*X_9_:*Relationship with Mother-in-law	Very satisfied (Reference) *X_91_* = 0, *X_92_* = 0, *X_93_* = 0, *X_94_* = 0
	No such relationship *X_91_* = 1, *X_92_* = 0, *X_93_* = 0, *X_94_* = 0
	Average *X_91_* = 0, *X_92_* = 0, *X_93_* = 1, *X_94_* = 0
	Satisfied *X_91_* = 0, *X_92_* = 0, *X_93_* = 0, *X_94_* = 1
*X_10_:*Personal Anxiety/Depression History	No=0, Yes=1
*X_11_:*Family Depression History	No=0, Yes=1
*X_12_:* Concern About Fetal Health	No=0, Yes=1
*X_13_:*Pregnancy Complications	No=0, Yes=1
*X_14_:*Fear of Childbirth	No=0, Yes=1
*X_15_:*Social Support	Entered as continuous raw value
*X_16_:*Coping Style	Entered as continuous raw value
*X_17_:*Vulnerable Personality	Entered as continuous raw value

According to the above variable assignment method, the independent variables with statistical differences in univariate analysis were entered into the regression equation.

The results showed that 10 variables were included in the regression model for the second and third trimesters: place of residence, monthly household income, recent appetite, mother-in-law and daughter-in-law relationship, history of anxiety and depression, obstetric complications during pregnancy, fear of childbirth, support utilization, active coping, and vulnerable personality. The model showed that women in rural areas during the second and third trimesters had a 3.26-fold higher risk of depression than those in urban areas. Higher monthly household income was associated with lower odds of positive depressive symptom screening. Pregnant women with average recent appetite had higher odds of positive depressive symptom screening than those with good appetite. Pregnant women with average mother-in-law and daughter-in-law relationships had higher odds of positive depressive symptom screening than those with satisfactory mother-in-law and daughter-in-law relationships. Pregnant women with a history of anxiety and depression had higher odds of positive depressive symptom screening.Pregnant women with greater fear of childbirth had higher odds of positive depressive symptom screening.Pregnant women with higher support utilization and active coping strategies had lower odds of positive depressive symptom screening. Pregnant women with higher vulnerable personality scores had higher odds of positive depressive symptom screening. See **Table 4** for details.

**Table 4:** Logistic regression model for depression in the second and third trimesters (n=588).

Variable	B	SE	β	*P*-value	OR	95% CI	
						Lower limit	Upper limit
Constant	-0.25	1.32	0.04	0.85	0.78		
Rural Residence	1.18	0.37	0.08	0.006	3.26	1.41	7.54
Monthly Income	-0.38	0.15	6.39	0.011	0.69	0.51	0.92
Average Appetite	0.77	0.24	10.33	0.001	2.15	1.35	3.43
Average MIL Relationship	1.24	0.38	10.66	0.001	3.47	1.64	7.31
No Anxiety/Depression History	-1.15	0.47	5.91	0.015	0.32	0.13	0.80
No Pregnancy Complications	-0.63	0.27	5.39	0.020	0.53	0.31	0.91
No Fear of Childbirth	-0.73	0.34	4.71	0.030	0.48	0.25	0.93
Support Utilization	-0.19	0.08	6.46	0.011	0.83	0.71	0.96
Positive Coping	-0.14	0.04	11.70	0.001	0.87	0.81	0.94
Vulnerable Personality	0.23	0.03	44.69	<0.001	1.26	1.18	1.35

### 3.4 Modeling results

**Figure 1:**
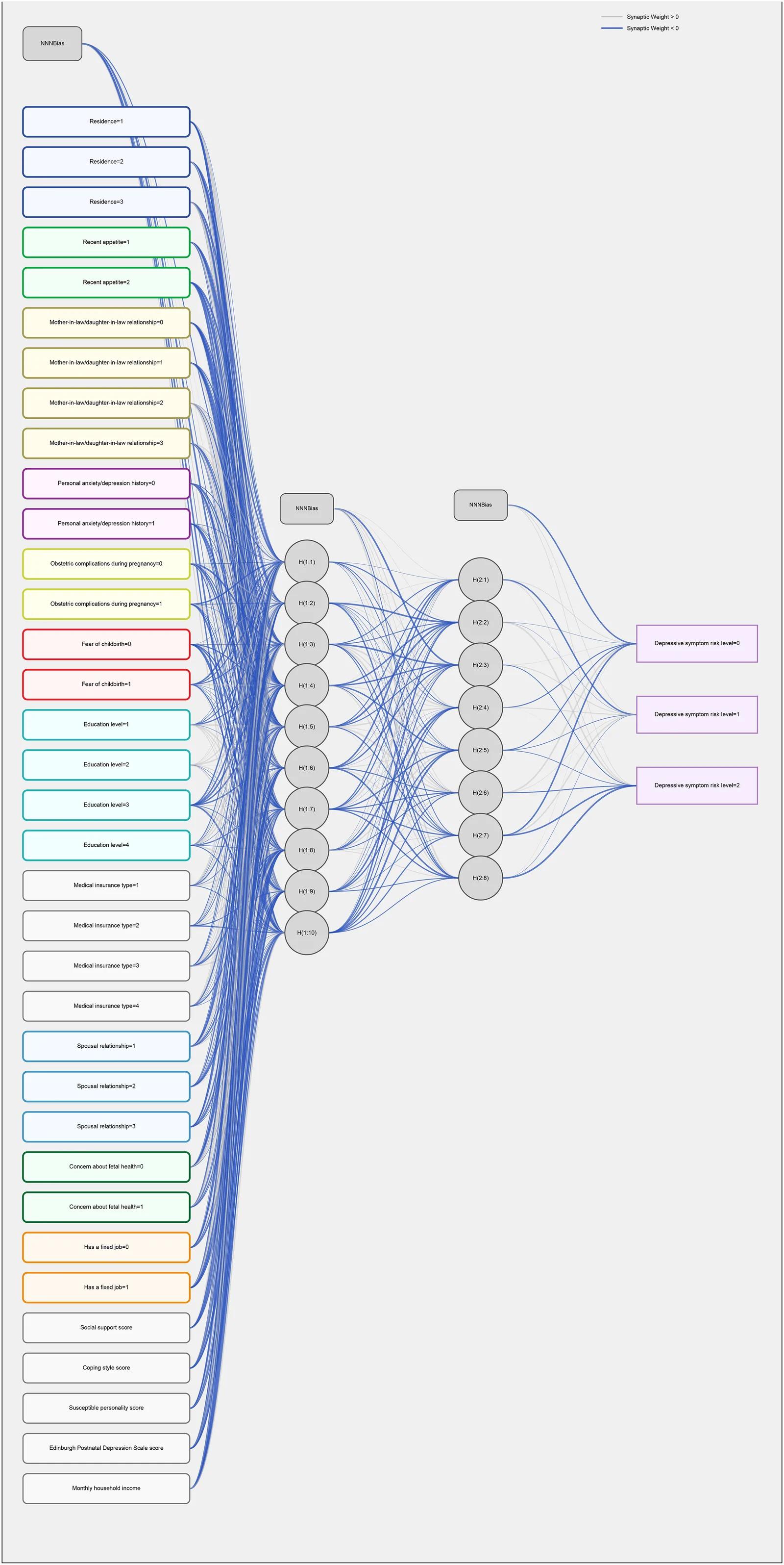
ANN model for predicting depression risk in the second and third trimesters.

**Figure 2:**
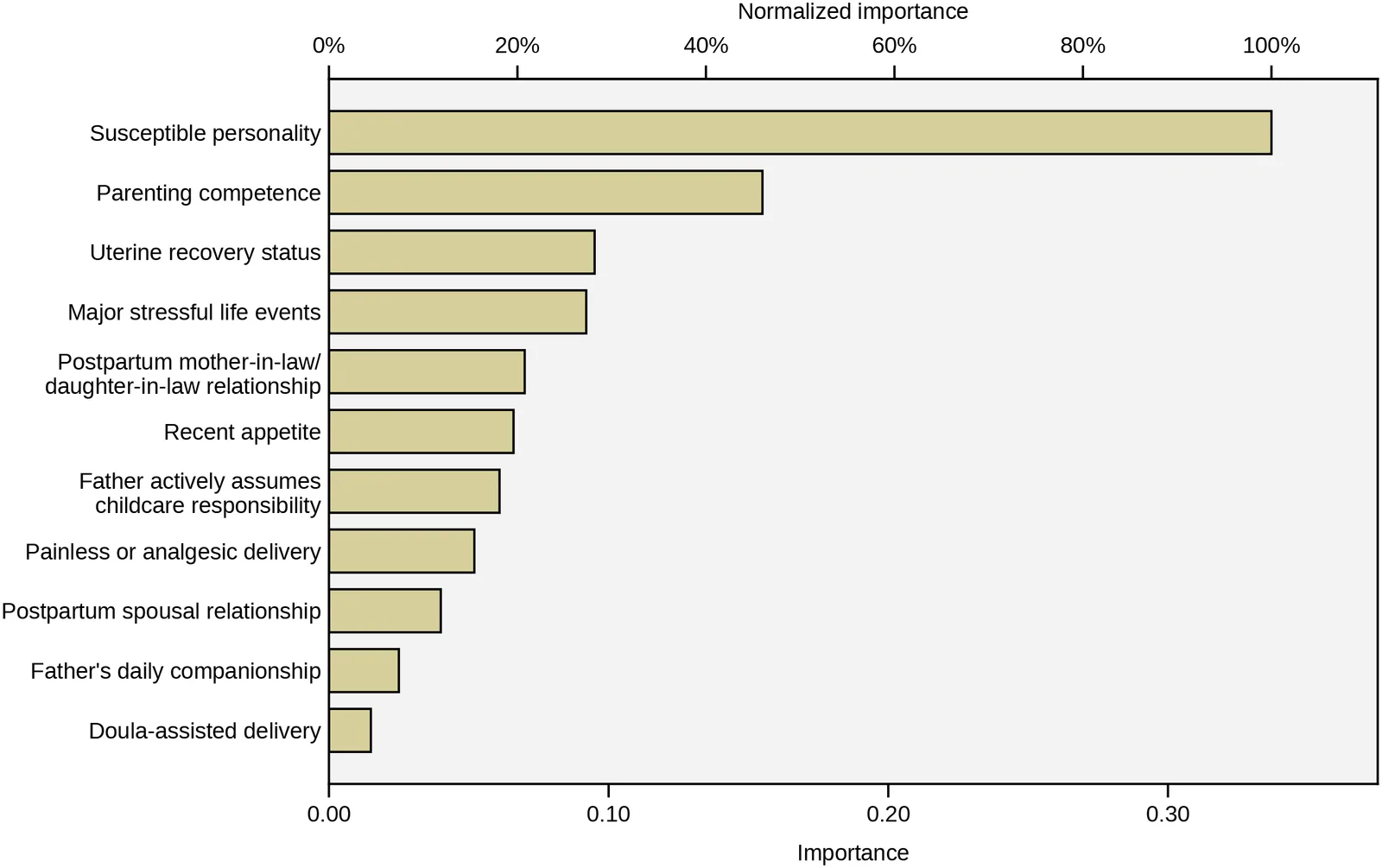
Importance of independent variables in the ANN model for predicting depression risk in the second and third trimesters.

An ANN model for predicting the risk of depression in the second and third trimesters: The machine ultimately selected 471 cases (80.1%) as the training set and 117 cases (19.9%) as the validation set. The input layer had 17 neurons, the first hidden layer generated 10 neurons, and the second hidden layer generated 8 neurons. The ANN model for predicting depression during mid- and late pregnancy is shown in **Figure 1**. The importance of the independent variables indicates that susceptible personality, social support level, and coping style are important factors contributing to depression during mid- and late pregnancy (see **Figure 2**).

**Figure 3:**
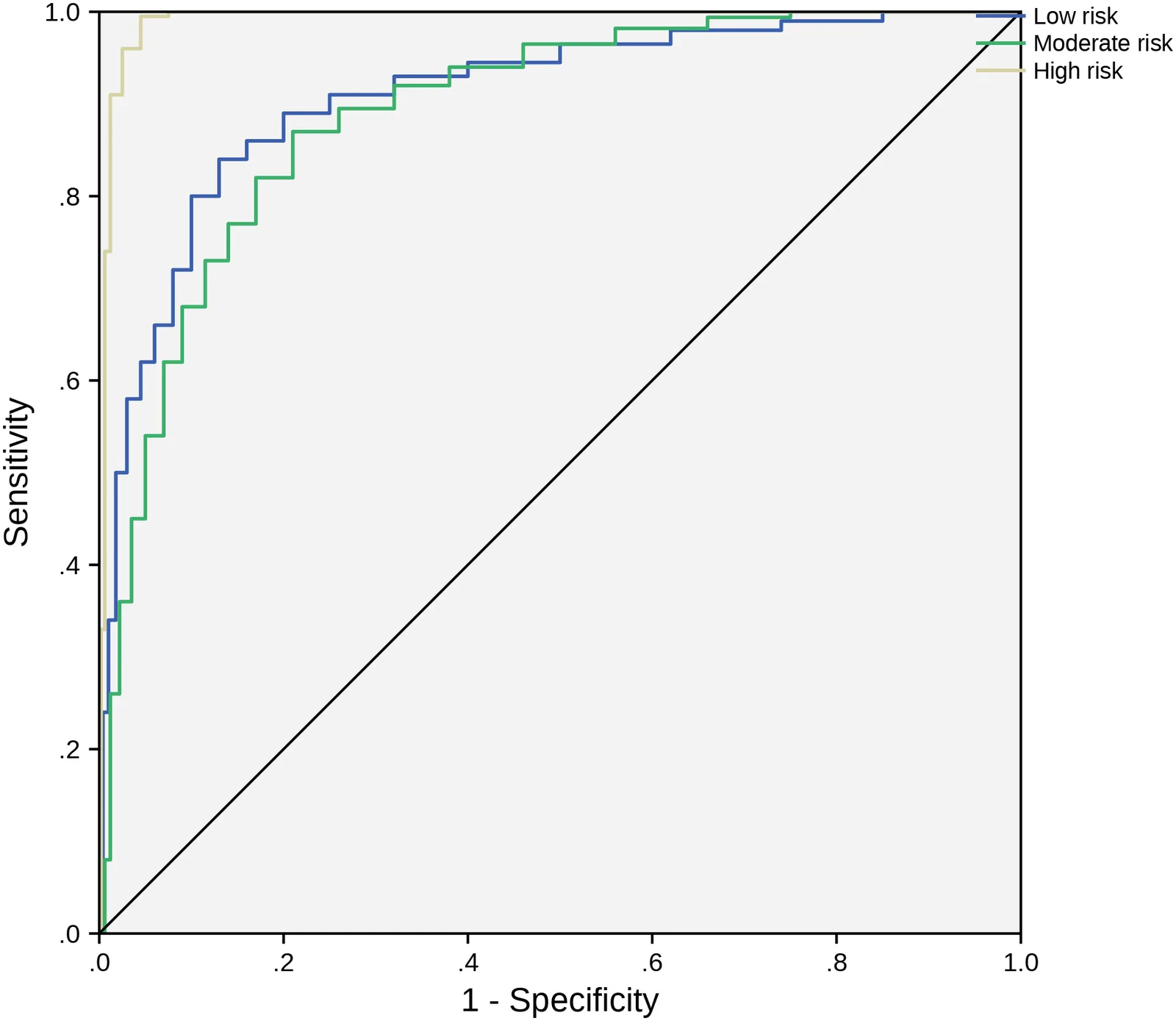
ROC curve of the ANN model for predicting depression risk in the second and third trimesters.

The ANN model for predicting mid-to-late pregnancy depression risk constructed in this study achieved accuracies of 94.1% and 86.9% during training and validation, respectively. The highest measured value was 1.58, with a split point of 0.5. The resulting validation set sensitivity was 0.97, specificity was 0.61, and the model’s AUC was 0.90. The ROC curve of the ANN model for predicting mid-to-late pregnancy depression is shown in **Figure 3**.

### 3.5 Evaluation of the effect of logistic regression model and ANN risk prediction model

Evaluation of the relevant indicators of the constructed logistic regression prediction model and ANN risk prediction model for the second and third trimesters revealed that both models demonstrated good predictive effectiveness. The ANN risk prediction model demonstrated satisfactory predictive effectiveness in screening perinatal women with true-positive depression, while the logistic regression model demonstrated good predictive effectiveness in screening perinatal women with confirmed negative depression. The effectiveness evaluation of the logistic regression model and the ANN risk prediction model is shown in **Table 5**.

**Table 5:** Evaluation of the effect of the Logistic regression model and the ANN risk prediction model in the second and third trimesters.

Model		Accuracy (%)	Sensitivity	Specificity	AUC
Second and third trimester	Logistic	79.6	0.74	0.85	0.87
	ANN	86.9	0.97	0.61	0.89

## 4 Discussions

### 4.1 Depression levels are higher in women during the second and third trimesters

This is higher than the depressive symptom detection rate reported by foreign scholars Lee (22) and Lara (23) and domestic scholars Wu Jiayan (24) and Liu Xiangxiang (25). The reasons may be: First,the perinatal depression detection standard used in this study is an EPDS score ≥9 points, and the depressive symptom detection rate obtained is higher than the detection rate obtained by EPDS score ≥13 points or Self-Rating Depression Scale (SDS) score ≥50 points. Second, this result may suggest that depressive symptoms among Chinese women in the second and third trimesters require greater attention. This may be related to the increasing work and life pressures of modern women and the trend of older pregnant women and increased childbearing pressure after the full liberalization of the three-child policy. Furthermore, since this study did not further employ different threshold values such as ≥10 or ≥13 for sensitivity analysis, the impact of different threshold values on the screening positivity rate and the stability of the model results still needs to be further verified in subsequent studies.

Secondly, the overall high level of depressive symptoms in the middle and late stages of pregnancy may be due to the following reasons: in the middle and late stages of pregnancy, due to the rapid growth of the fetus and the enlargement of the uterus, the pregnant woman gains weight rapidly, which may be associated with anxiety and lack of confidence due to body changes, and also be related to the sleep quality and normal interpersonal communication of pregnant women (26). Therefore, the detection rate of depressive symptoms in this period is higher.

### 4.2 Factors influencing depression during the second and third trimesters

The results in **Table 1** show that depressive symptoms in the second and third trimesters were associated with 13 factors, among which the history of anxiety and depression and fear of childbirth are two factors that deserve attention. Women in the second and third trimesters who have a history of anxiety and depression have a higher risk of PND, which is similar to the results of Xiao Ao Shuang’s study (27). The reasons may be: on the one hand, changes in hormone levels may be related to the recurrence of depressive symptoms;on the other hand, pregnancy and childbirth are external events that stimulate women with a history of anxiety and depression, making them more likely to.experience depressive symptoms. For perinatal women with a history of anxiety and depression, clinical nurses should pay more attention to changes in their mental state and guide them to relieve stress through breathing exercises, music therapy, aromatherapy, etc.; or provide them with psychological assistance channels to help them seek professional help.

The proportion of women with EPDS scores ≥ 9 who have a fear of childbirth in the middle and late stages of pregnancy is higher than that of women without a fear of childbirth. Childbirth is an intense physical and mental experience for women. Fear of the pain of childbirth, concerns about unsuccessful childbirth, and exposure of privacy may make pregnant women feel fear, anxiety, and tension. The fear becomes more intense in the late stages of pregnancy, and they may even be unwilling to face childbirth. A study involving 500,000 mothers in Finland showed that pregnant women who are afraid of childbirth during pregnancy have higher odds of depressive symptoms. Studies have confirmed (28) that the degree of childbirth fear is significantly associated with the occurrence of postpartum depression, and it also is associated with a higher incidence of adverse events during childbirth, and even endangers the health of mothers and newborns. Therefore, for pregnant women with a fear of childbirth, obstetric clinic staff need to intervene in a timely manner. It is recommended that pregnant women and their spouses be provided with knowledge about childbirth during routine prenatal examinations and their doubts be answered in a timely manner (29). Experienced midwives can provide pregnant women with answers to pregnancy and childbirth knowledge and professional training, including exercise, childbirth skills, pain relief during childbirth, diet during childbirth, postpartum recovery, and newborn care. “Pregnant mother childbirth lectures” can also be held to invite mothers who have had successful childbirth to share their experiences. Before delivery, experienced midwives can teach delivery techniques and, if necessary, conduct delivery rehearsals (30) to familiarize pregnant women with the delivery process and alleviate their panic caused by fear of delivery.

### 4.3 Important factors affecting depression in the second and third trimesters of pregnancy

According to the results in **Table 4**, rural residence, recent average appetite, average relationship between mother-in-law and daughter-in-law, and susceptible personality are risk factors for depressive symptoms in the second and third trimesters of pregnancy, while high monthly family income, no history of anxiety and depression, no obstetric complications during pregnancy, no fear of childbirth, high utilization of support, and active coping strategies are factors associated with lower odds of depressive symptoms.

Among them, women who live in rural areas and have an average relationship with their mother-in-law and daughter-in-law have the highest odds of depressive symptoms in the middle and late stages of pregnancy. This is consistent with the research results of Hu (7) and Sarah (31). The reasons for this may be as follows: ① The proportion of women who live in rural areas with an EPDS score of ≥9 is the highest, at 53.8%, followed by those in urban areas, and the lowest in urban areas. The reasons for this may be as follows: First, the inherent rural mentality of favoring sons over daughters may make rural women feel constrained during pregnancy; second, the communication and emotional needs of rural women in the middle and late stages of pregnancy are easily ignored and restricted; third, because rural medical conditions are relatively backward compared to urban areas, many pregnant women may not have regular prenatal checkups, let alone screening for the mental health of women in the middle and late stages of pregnancy. The recognition rate of depression is low, and they may have limited access to timely mental health assistance. Therefore, obstetric clinic staff can recommend that they choose hospitals with good medical conditions for regular prenatal checkups, listen patiently and explain, and provide psychological assistance resources when necessary. ② Pregnant women with an average relationship between their mother-in-law and daughter-in-law have higher odds of depressive symptoms than those with a satisfactory relationship between their mother-in-law and daughter-in-law. This survey shows that 56.1% of pregnant women live with their parents-in-law. The harmonious relationship between mother-in-law and daughter-in-law is closely linked to the pregnant woman’s mood. Fluctuations in hormone levels and differences in lifestyle habits may also be associated with depressive symptoms.Long-term negative mother-in-law-daughter-in-law relationships may be associated with aggravated depressive symptoms. Therefore, obstetric outpatient caregivers should pay special attention to pregnant women from rural areas, those with poor mother-in-law-daughter-in-law relationships, and those with sensitive personalities.

**Figure 2** shows that vulnerable personality, social support level, coping style, etc. are important factors associated with depressive symptoms in the middle and late stages of pregnancy, which is consistent with the research results of Yang Kuanhong, Dennis, and Zhang Liu. Yang Kuanhong believes (32) that people with sensitive personalities are more likely to evaluate external events as stressors, produce stronger stress reactions, and may generate and accumulate more negative emotions, while people with extroverted and friendly personalities may take a more positive attitude towards events, so their d odds of depressive symptoms may be lower. For perinatal women with vulnerable personalities, nursing staff should establish a good nurse-patient relationship, understand their nursing needs and psychological needs, and provide personalized and humane perinatal care; pay attention to their attitudes, words, and behaviors, listen patiently, answer their questions seriously, and perceive their emotional changes.

Social support is considered to be the key to understanding psychological adaptation and psychological maladjustment (33). When individuals are exposed to stressful events, social support may buffer the adverse effects of potential stressful events. When perinatal women experience depression or encounter stressful events, they can seek help from relatives, friends, colleagues or organizations in a timely manner and actively talk to gain support. Therefore, it is recommended that nursing staff communicate with those who accompany prenatal checkups or primary caregivers and pay more attention to their emotional changes; at the same time, they should actively respond to the needs of perinatal women and jointly help perinatal women establish an effective social support system so that they can feel the help of the social support system and thus help alleviate negative emotions in a good social support system.

Alves et al. (34) believe that perinatal women can reduce the negative impact of labor stress by facing stressful events in a positive and optimistic manner, while adopting negative coping strategies such as escaping and giving up may be less effective in dealing with stressful events and may be associated with confusion, negativity, and pessimism, which in turn may be associated with depressive symptoms. It is recommended that nursing staff guide perinatal women to actively cope with stressful events. This can subtly influence pregnant women’s cognition of external pressure through health education, guide them to look at problems from different perspectives, and inform them that blindly avoiding and negatively coping will not solve the problem and may further aggravate the situation. Reasonable suggestions can be given from a psychological perspective (35, 36).

### 4.4 Evaluation of the effectiveness of the depressive symptom prediction model in the second and third trimesters

**Table 5** shows that both prediction models have good predictive performance and each has its own advantages. Both models can be used for early screening and risk identification of perinatal depressive symptoms. The perinatal depressive symptom prediction model based on logistic regression can provide a basis for clinical nurses to identify factors associated with positive depressive symptom screening. Compared with the logistic regression model, the perinatal depressive symptom prediction model based on ANN has higher sensitivity but lower specificity, indicating that ANN is more advantageous in identifying individuals with potential depressive symptoms. It can help nurses identify high-risk groups for perinatal depressive symptoms and provide an objective and reliable tool for screening perinatal depressive symptoms.

## 5 Summary

In this study, depressive symptom levels among women in the second and third trimesters were high and were associated with multiple factors. Obstetric outpatient nursing staff can develop targeted measures based on this model to prevent the occurrence and development of depression.

This study has limitations: the cross-sectional survey of the positive rate of depressive symptom screening in women in the mid-to-late stages of pregnancy and its associated factors cannot reflect the continuity of changes in individual psychological states. Future studies will establish multiple observation points before and after delivery to continuously track the psychological state of women during the perinatal period, and will increase the sample size and measure associated factors to improve the model. In subsequent studies, this model will be applied to depressive symptom screening and prediction in perinatal women for external validation, to evaluate the model’s actual predictive effectiveness, and an attempt will be made to translate the model’s findings into practical applications.

## Data Availability

The original contributions presented in the study are included in the article/supplementary material. Further inquiries can be directed to the corresponding author.
