# Correction: Research on the construction of prediction model for depressive symptom in the second and third trimester of pregnancy based on artificial neural network

**DOI:** 10.3389/fpsyt.2026.1926517

**Published:** 2026-08-10

**Authors:** Liuyue Wang, Dandan Zhou, Yanhui Liu, Zhiqun Liu, Huan Wan

**Affiliations:** 1 Nursing Department, The First Affiliated Hospital of Hunan Normal University (Hunan Provincial People’s Hospital), Changsha, Hunan, China; 2 Nursing College, School of Medicine, Hunan Normal University, Changsha, Hunan, China; 3 Kiang Wu Nursing College of Macau, Macao, SAR, China; 4 School of Nursing, Changsha Medical College, Changsha, Hunan, China; 5 Nursing Department, Hunan Provincial People’s Hospital (The First Affiliated Hospital of Hunan Normal University), Changsha, Hunan, China; 6 Department of Emergency Medicine, Clinical Research Center for Emergency and Critical Care in Hunan Province, Hunan Provincial Institute of Emergency Medicine, Hunan Provincial Key Laboratory of Emergency and Critical Care Metabonomics, Hunan Provincial People’s Hospital, The First Affiliated Hospital of Hunan Normal University, Changsha, Hunan, China; 7 Department of Psychology, College of Education Science, Hunan Normal University, Changsha, Hunan, China

**Keywords:** artificial neural network, depression, pregnancy, psychological nursing, risk prediction

Affiliation “Nursing Department, The First Affiliated Hospital of Hunan Normal University (Hunan Provincial People’s Hospital), Changsha, Hunan, China” was erroneously omitted and has been added to author “Liuyue Wang”.

The original version of this article has been updated.

